# Activation of the Nrf2 Signaling Pathway by a Ginseng–Salvia Root–Notoginseng Composite Alleviates Ulcerative DSS-Induced Colitis via Restoring Gut Microbiota and the Intestinal Barrier

**DOI:** 10.3390/antiox15030320

**Published:** 2026-03-04

**Authors:** Xinao Lyu, Liurong Zhang, Jia Si, Shasha Dai, Huaiyu Su, Shuhuan Lyu, Lin Chen, Jianwei Sun, Xiangqun Jin, Haiyan Li

**Affiliations:** 1 Department of Pharmacy, Jilin University, Changchun 130021, China; lvxa24@mails.jlu.edu.cn (X.L.); zhanglr24@mails.jlu.edu.cn (L.Z.); jiasi23@mails.jlu.edu.cn (J.S.); daiss24@mails.jlu.edu.cn (S.D.); suhy24@mails.jlu.edu.cn (H.S.); lvsh25@mails.jlu.edu.cn (S.L.); chenlin25@mails.jlu.edu.cn (L.C.); sunjw25@mails.jlu.edu.cn (J.S.); 2 Department of Pharmacy, Changchun University of Chinese Medicine, Changchun 130021, China

**Keywords:** DSS-induced colitis, Nrf2 signaling pathway, intestinal barrier function, gut microbiota dysbiosis, tight junction proteins, multi-target therapy

## Abstract

Current treatments for ulcerative colitis (UC) often fail to adequately address its multifactorial pathogenesis, which involves oxidative stress, barrier dysfunction, and gut microbiota dysbiosis. This study evaluated the therapeutic potential and multi-targeting mechanism of a ginseng, salvia root, and notoginseng oral solution (GSNS) in a mouse model of colitis induced by dextran sulfate sodium (DSS). Based on high-performance liquid chromatography-tandem mass spectrometry (HPLC-MS/MS) technology, 25 major bioactive components were identified. Following the induction of colitis with 3.5% DSS in C57BL/6J mice, the animals were treated with the GSNS (40, 80, or 160 mg/kg/day) or 5-Amino Salicylic Acid (5-ASA). The therapeutic efficacy was assessed via disease activity, histopathological staining, cytokines and oxidative stress analysis, and a barrier integrity test. Combined data from Western blot, qPCR, immunohistochemistry, electron microscopy, and 16S rRNA sequencing indicate that the therapeutic effect of the GSNS against colitis is attributable to its dual role in dampening pro-inflammatory cytokines and potentiating antioxidant defenses via the Nrf2/HO-1 signaling pathway. It also upregulated Occludin expression, repaired tight junctions, and was associated with beneficial alterations in the gut microbiota, as evidenced by increased Prevotellaceae and suppressing *Escherichia-Shigella*. These findings demonstrated that the GSNS exerts a multi-target effect against colitis by synergistically enhancing antioxidant defense, repairing the intestinal barrier, and modulating microbial ecology, supporting its potential as a promising natural compound-based candidate for DSS-induced colitis treatment.

## 1. Introduction

Ulcerative colitis (UC) is a chronic, relapsing inflammatory bowel disease. The core pathology of UC involves disruption to the epithelial barrier, leading to diffuse mucosal injury [1]. Its pathogenesis involves a vicious cycle comprising multiple components, such as oxidative stress, uncontrolled inflammatory responses, and intestinal dysbiosis [2,3,4]. Current therapeutic strategies primarily target specific inflammatory pathways but often fail to address underlying issues such as persistent barrier dysfunction, impaired endogenous antioxidant defenses, and gut microbiota dysbiosis [5,6,7]. This can lead to incomplete remission, high relapse rates, and a suboptimal treatment response in some patients. Consequently, the development of multi-target therapeutic strategies that can intervene in multiple key pathological links of UC simultaneously has become urgent.

Natural products, with their multi-component nature, offer a promising avenue for intervening in this complex pathological network [8]. A classic formulation in traditional Chinese medicine composed of ginseng (*Panax ginseng*), salvia root (*Salvia miltiorrhiza*), and notoginseng (*Panax notoginseng*) has a long history of clinical application in traditional Chinese medicine and holds an important position in both theoretical and clinical practice. Key bioactive compounds derived from these herbs, including ginsenosides, salvianolic acids, and notoginsenosides, have demonstrated anti-inflammatory, antioxidant, gut microbiota-modulating, and intestinal barrier-repairing properties [9,10,11].

While the individual pharmacology of ginseng, salvia root, and notoginseng has been investigated, their combined effects in UC remain unclear [12,13,14,15]. Recent research has largely focused on single herbs or isolated compounds, lacking a systematic assessment of the overall therapeutic efficacy of the ginseng, salvia root, and notoginseng extract in UC [16,17,18]. Consequently, the material basis and integrated mechanisms underlying its potential multi-component synergistic effects are still insufficiently clarified. Moreover, most existing mechanistic investigations are limited to single pathways (e.g., anti-inflammation) and fail to incorporate the interplay between key processes such as Nrf2-mediated antioxidant signaling, intestinal barrier repair, and gut microbiota modulation into a unified framework. A holistic, network-based understanding of the formula’s therapeutic actions is thus impeded by the current fragmented approach. At the microbiome level, current evidence is predominantly characterized by compositional descriptions, with a lack of functional validation linking specific microbial shifts, key taxa, or their dynamic interactions to therapeutic outcomes [19]. Therefore, the scientific rationale for using this traditional Chinese medicine (TCM) compound in UC remains incomplete, hindering both systematic formula optimization and the development of precise, mechanism-informed clinical applications.

This study developed the ginseng, salvia root, and notoginseng oral solution (GSNS) and evaluated its therapeutic potential against UC in a DSS-induced colitis mice model. The chemical profile of the GSNS was characterized by LC-MS/MS. Therapeutic efficacy was assessed by monitoring body weight, calculating disease activity indices, measuring colon length, and performing histopathological assessment. To investigate the underlying mechanisms, the expression and localization of inflammatory cytokines, oxidative stress markers, key proteins/genes in the Nrf2 signaling pathway, and tight junction proteins were quantified using enzyme-linked immunosorbent assay (ELISA), Western blot, qPCR, and immunohistochemistry (IHC). In parallel, 16S rRNA gene sequencing was performed, combined with diversity analysis, linear discriminant analysis effect size (LEfSe), differential species screening, and correlation network analysis, to systematically elucidate the regulatory effects of the GSNS on gut microbiota structure and microbial interaction networks. Collectively, these results demonstrate that the GSNS alleviates DSS-induced colitis through multi-target mechanisms involving the reduction of oxidative stress, repair of the intestinal barrier, and regulation of gut microbiota ecology.

## 2. Materials and Methods

### 2.1. Primary Chemical Reagents

The herbal materials (*Panax ginseng*, *Salvia miltiorrhiza*, and *Panax notoginseng*) were purchased from Jilin Large Pharmacy Pharmaceutical Co., Ltd. (Changchun, China). Dextran sulfate sodium (DSS; molecular weight: 40,000 Da, catalog no. BD123894) was purchased from Bide Pharmatech Co., Ltd. (Beijing, China). Occludin antibody (1:500, BSM-61062R) was purchased from Bioss (Beijing, China). Antibodies against Nrf2/NFE2L2 (66504-1-Ig), HO-1/HMOX1 (66743-1-Ig), GAPDH (66004-1-Ig), and Lamin B1 (66095-1-Ig) were obtained from ProteinTech (Wuhan, China). ELISA kits for interleukin-6 (IL-6), interleukin-10 (IL-10), interleukin-1β (IL-1β), myeloperoxidase (MPO), tumor necrosis factor-α (TNF-α), malondialdehyde (MDA), glutathione (GSH), and superoxide dismutase (SOD) were purchased from FeiYa Biotechnology Co., Ltd. (Nanjing, China) and Multi Sciences (Hangzhou, China). TRIzol reagent was obtained from Invitrogen (Carlsbad, CA, USA). RIPA lysis buffer (containing PMSF; catalog no. P0013B), phenylmethylsulfonyl fluoride (PMSF; catalog no. ST505), and a bicinchoninic acid (BCA) protein assay kit (catalog no. P0012) were purchased from Beyotime Biotechnology (Shanghai, China). A polyvinylidene fluoride (PVDF) membrane (catalog no. IPVH00010) was obtained from Millipore (Burlington, MA, USA). The 16S rRNA gene (V4 region) Amplification and Library Preparation Kit (catalog no. 12933ES96) was purchased from Yeasen Biotechnology Co., Ltd. (Shanghai, China). The following reagents and kits were purchased from Servicebio Biotechnology Co., Ltd. (Wuhan, China): Magnetic Bead-Based Bacterial Genomic DNA Extraction Kit, Universal Tissue Fixative, Optimal Cutting Temperature (OCT) Embedding Medium, Hematoxylin and Eosin (H&E) Staining Kit, RNA Extraction Reagent, SweScript All-in-One RT SuperMix, and 2 × Universal Blue SYBR Green qPCR Master Mix. Sequencing was performed on the DNBSEQ-G99 platform by Shenzhen BGI Intelligent Manufacturing Technology Co., Ltd. (Shenzhen, China).

### 2.2. Preparation of Ginseng, Salvia Root, and Notoginseng Oral Liquid

The compound extract GSNS was prepared using a standardized reflux extraction protocol. Briefly, authenticated crude drugs of ginseng, salvia root, and notoginseng were mixed in a 1:1:1 weight ratio. This ratio refers to the proportion of raw materials used in the preparation process and does not represent the quantitative composition of individual components in the final extract. The mixture underwent two rounds of reflux extraction with 50% ethanol aqueous solution (10:1, *v*/*w*), each for 3 h. The combined extracts were filtered, and the filtrate was concentrated under reduced pressure at 40–50 °C using a rotary evaporator. The resulting concentrated liquid was then lyophilized to a constant weight, yielding the dry extract powder. The extraction yield was calculated accordingly. For in vivo administration, a precise weight of the lyophilized powder was reconstituted in sterile distilled water to the desired concentration. The solution was sterilized by filtration through a 0.22 µm membrane and aliquoted for storage at −20 °C.

### 2.3. Chemical Profiling of GSNS Using UPLC-MS/MS

UPLC-MS/MS analysis was performed on a Dionex UltiMate 3000 system (Thermo Fisher Scientific, Germering, Germany) coupled to a Thermo Q-Exactive Plus mass spectrometer (Thermo Fisher Scientific, Bremen, Germany). to determine the chemical constituents of the GSNS according to a published method [20]. Chromatographic separation utilized an ACQUITY UPLC HSS T3 column (150 × 2.1 mm, 1.8 μm). The mobile phase consisted of (A) deionized water containing 0.1% formic acid and (B) acetonitrile containing 0.1% formic acid. These were delivered in gradient elution mode as follows: 0–5 min: 5% B; 5–95 min: 5% to 95% B (linear gradient); 95–100 min: 95% B. Thereafter, the system was returned to the initial conditions (95% A, 5% B) within 0.1 min, after which it was equilibrated for 10 min. A constant flow rate of 0.3 mL/min was maintained throughout the run. Data acquisition and processing were carried out using Xcalibur and TraceFinder 5.1 software (Thermo Fisher Scientific, Waltham, MA, USA).

### 2.4. Animals and Experimental Design

Male C57BL/6J mice weighing 20 ± 2 g were obtained from Speifu (Beijing) Biotechnology Co., Ltd. (Changchun, China; license no. SYXK (Ji) 2021-0003). After a three-day acclimatization period under controlled conditions (25 ± 2 °C, 60 ± 5% relative humidity, 12 h light/dark cycle), mice judged to be healthy based on normal activity, glossy fur, and no visible signs of disease were randomly divided into six groups (*n* = 6) using a computer-generated randomization sequence. Blinding was implemented for outcome assessment and data analysis. To minimize confounders, cage positions were rotated daily, and treatments were administered at the same time each day (9:00 AM). The control group received normal drinking water. The model group received 3.5% (w/v) DSS in drinking water for 7 days. The positive control group received 3.5% DSS water plus 5-ASA (100 mg/kg/day) by oral gavage. Three treatment groups (GSNS_L_, GSNS_M_, GSNS_H_) received 3.5% DSS water plus the GSNS at doses of 40, 80, and 160 mg/kg/day, respectively, via oral gavage. Throughout the 7-day experimental period (days 0–7), all treatments were administered daily. On day 7, all mice were euthanized. Peripheral blood was collected via orbital bleeding, and colon tissues and intestinal contents were immediately harvested for subsequent analysis (see experimental timeline in figure in Section 3.). Animal experiments used six mice per group (*n* = 6) based on pilot study effect sizes for sufficient statistical power. For molecular analyses (Western blot, qPCR), three randomly selected samples per group (*n* = 3) served as biological replicates and were sufficient to validate in vivo mechanisms, consistent with the Reduction principle of the 3R framework [21]. All procedures were approved by the Animal Care and Use Committee of the School of Pharmacy, Jilin University (approval no. 20250121). To minimize pain and distress, anesthesia (isoflurane or pentobarbital sodium) was used during colitis induction and euthanasia, and animals were housed under SPF conditions with soft bedding and free access to food and water. Expected adverse events related to colitis induction (e.g., weight loss, diarrhea, bloody stool) were observed, with no unexpected events. Humane endpoints included body weight loss > 20%, disease activity index score ≥ 10 for two consecutive days, a moribund state, or inability to access food/water for >24 h. Animals were monitored twice daily; body weight and DAI were recorded daily. No animals reached humane endpoints before scheduled euthanasia.

### 2.5. Mouse Body Weight, Disease Activity Index, and Colon Length

Body weights were recorded for all mice groups each day before treatment; fecal samples were simultaneously evaluated for consistency and signs of bleeding. The disease activity index (DAI) score provides a comprehensive reflection of disease severity in the UC model mice. The calculation formula is DAI = (Body Weight Loss Score + Fecal Consistency Score + Blood in Feces Score)/3 [22]. The colon was photographed and its length measured to assess the degree of inflammatory response.

### 2.6. Analysis of Serum and Colon Cytokines and Oxidative Stress Markers

Blood samples were processed by centrifugation at 4000 rpm for 20 min at 4 °C to obtain the serum. Colon samples were homogenized in PBS and centrifuged at 13,000 rpm for 10 min at 4 °C to collect the supernatant. Commercial ELISA kits were used to quantify the levels of cytokines (TNF-α, IL-6, IL-10, and IL-1β) in both the serum and the colonic supernatant and the levels of oxidative stress markers (MPO, MDA, SOD, and GSH) in the colonic supernatant according to the manufacturers’ instructions.

### 2.7. Hematoxylin–Eosin Staining and Immunohistochemical Analysis of Mouse Colon Tissue

Histopathological assessment was conducted based on H&E staining. After routine processing (fixation in 4% paraformaldehyde, paraffin embedding, and sectioning), tissue sections were scored for pathological changes using an established criterion [23]. To detect Nrf2 using IHC, the paraffin sections were subjected to antigen retrieval. Subsequently, endogenous peroxidase was quenched with 3% CH_3_OH-H_2_O_2_ and non-specific sites were blocked with 3% bovine serum albumin (BSA). The sections were then incubated with an anti-Nrf2 primary antibody overnight at 4 °C in a humidified chamber, followed by incubation with a horseradish peroxidase (HRP)-conjugated secondary antibody. Signal visualization was achieved using 3,3′-diaminobenzidine (DAB) as the chromogen, and the development time was monitored under a microscope. Finally, the sections were counterstained with hematoxylin, dehydrated, cleared, and mounted. Protein expression of Nrf2 was quantified using the H-Score method. This semi-quantitative scoring system evaluates both staining intensity and the proportion of positive cells. The staining intensity was graded on a scale of 0 to 3 (0, negative; 1, weak/pale yellow; 2, moderate/brownish yellow; 3, strong/dark brown). The H-Score was calculated using the formula $H-Score=\sum \left(pi\times i\right)$, where ‘i’ is the intensity score (0–3) and ‘pi’ is the percentage of cells stained at that intensity [24].

### 2.8. Ultrastructural Analysis of Mouse Colon via Transmission Electron Microscopy

Following initial fixation in 4% glutaraldehyde, the colon tissues were prepared for transmission electron microscopy (TEM). This involved sectioning, dehydration, embedding, polymerization, and ultrathin sectioning (4 µm). The sections were then post-stained with uranyl acetate and lead citrate, after which they were observed under a Hitachi HT7700 TEM (Hitachi High-Technologies Corporation, Tokyo, Japan).

### 2.9. RNA Extraction and Real-Time Fluorescent Quantitative PCR Analysis

Total RNA was extracted from colon tissue using TRIzol reagent and precipitated with isopropanol. Total RNA was first quantified for concentration and purity on a NanoDrop 2000 Spectrophotometer (Thermo Fisher Scientific, USA). Subsequently, 1 µg of RNA was reverse-transcribed into cDNA using the SweScript All-in-One SuperMix for qPCR Kit (Servicebio, China) following the kit protocol. Quantitative PCR was then carried out on a StepOnePlus^TM^ System (Applied Biosystems, Waltham, MA, USA) with Universal Blue SYBR Green qPCR Master Mix (Servicebio, China). After an initial 30 s step at 95 °C, samples underwent 40 cycles of denaturation (95 °C, 15 s) and annealing/extension (60 °C, 30 s). Specificity was assessed by melting curve analysis. Gene expression was quantified relative to the geometric mean of Ywhaz and Hprt1 using the 2^−^^∆∆Ct^ method. For miRNA analysis, miR-146a-5p was normalized against U6 as the reference gene. Primer sequences are listed in Table 1.

### 2.10. Protein Expression Analysis of Relevant Signaling Pathways (Western Blot)

Total protein was extracted from colon tissues with RIPA lysis buffer supplemented with PMSF, and concentrations were quantified using a BCA assay kit. Equal protein aliquots were then separated by SDS-PAGE and electrophoretically transferred onto PVDF membranes. Following blocking, the membranes were probed overnight at 4 °C with primary antibodies against Occludin, Nrf2, HO-1, Lamin B1, and GAPDH. GAPDH and Lamin B1 served as loading controls for cytoplasmic and nuclear proteins, respectively. After washing, the blots were incubated with horseradish peroxidase (HRP)-conjugated secondary antibodies for 1 h at room temperature. Band detection was performed with a JUNYI chemiluminescent imager. Quantitative analysis was carried out by measuring the optical density of each band using Image J software (NIH, Version 1.8).

### 2.11. Microbial Community Analysis Based on the 16S rRNA Gene

The resulting amplicons were purified, pooled in equimolar amounts, and prepared for sequencing library construction. Sequencing was performed on the DNBSEQ-G99 platform (2 × 300 bp paired-end), yielding approximately 50,000 raw reads per sample. Data processing was conducted within the QIIME2 (v2023.5) workflow. Processing of the raw 16S rRNA sequencing data involved demultiplexing, quality filtering, merging, and denoising using DADA2, followed by clustering into amplicon sequence variants (ASVs). Taxonomic annotations for the ASVs were derived from the SILVA 138 database. Subsequent analyses included α-diversity metrics (Chao1, Simpson, Shannon, richness, Pielou and observed ASVs), a β-diversity analysis via PCoA based on Bray–Curtis and Jaccard distances, an examination of ASV sharing using Venn diagrams, and a profiling of community composition at phylum, genus, and species levels, supplemented by a phylogenetic tree. LEfSe was applied to identify key differential microbial features (LDA score > 3.5), listing the top 30 most significant taxa. Finally, a species abundance-based correlation network was constructed. This integrated analysis provided a systematic characterization of DSS-induced colitis and the impact of GSNS intervention on the structure of the murine gut microbiota.

### 2.12. Statistical Analysis

Statistical analysis was performed using GraphPad Prism 10.0 (GraphPad Software, San Diego, CA, USA) and SPSS Statistics 22.0 (lBM, Armonk, NY, USA). All data are presented as mean ± standard error of the mean (SEM) from at least three independent experiments. For comparisons among multiple groups, one-way analysis of variance (ANOVA) followed by Tukey’s post hoc test was conducted using SPSS Statistics 22.0. For non-parametric data (including disease activity scores and histological scores), the Kruskal–Wallis test followed by Dunn’s multiple comparison test was performed. Statistical significance was set at *p* < 0.05, and significance levels are indicated as follows: * *p* < 0.05, ** *p* < 0.01, *** *p* < 0.001.

## 3. Results

### 3.1. LC-MS/MS-Based Identification of Bioactive Constituents in GSNS

**Figure 1:**
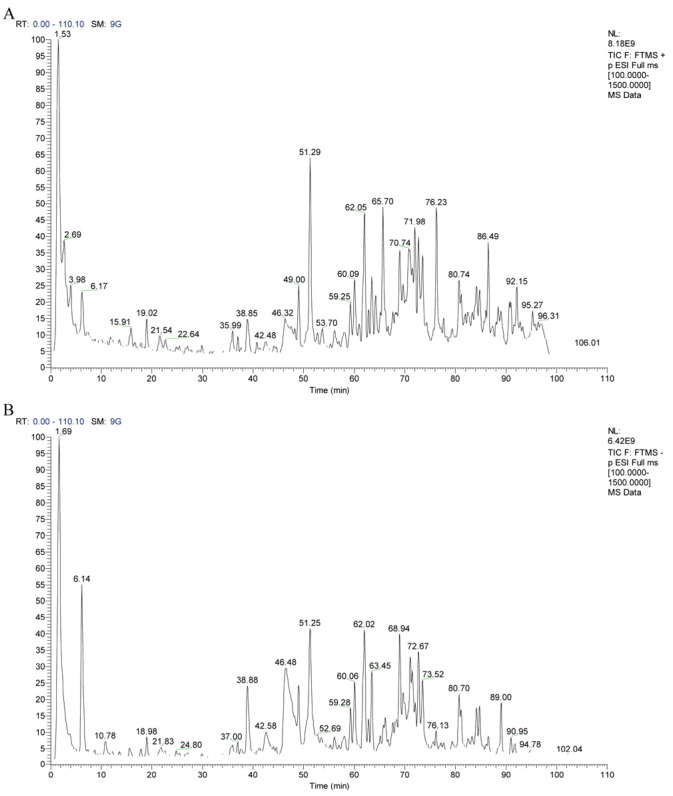
Total ion chromatography (TIC) of the GSNS extract. (**A**) Profile obtained in positive ion mode. (**B**) Profile obtained in negative ion mode.

The chemical profile of the GSNS was established by LC-MS/MS analysis. A total of 25 major bioactive components were identified by comparing retention times and mass spectra with authenticated reference standards (see the total ion chromatogram in Figure 1). These components included various ginsenosides (e.g., 20(R)-ginsenoside Rg2, ginsenoside Rb1 and Rg1) [25,26,27], notoginsenosides (e.g., notoginsenosides R1), and salvianolic acids (e.g., salvianolic acid B) [28,29]. Identification criteria were set at a mass error tolerance of <3 ppm and an identification score of >80 [30]. Detailed data for each compound are summarized in Table 2, thereby elucidating the material basis underlying the pharmacological efficacy of the GSNS.

### 3.2. Impact of GSNS on DAI and Associated Metrics

**Figure 2:**
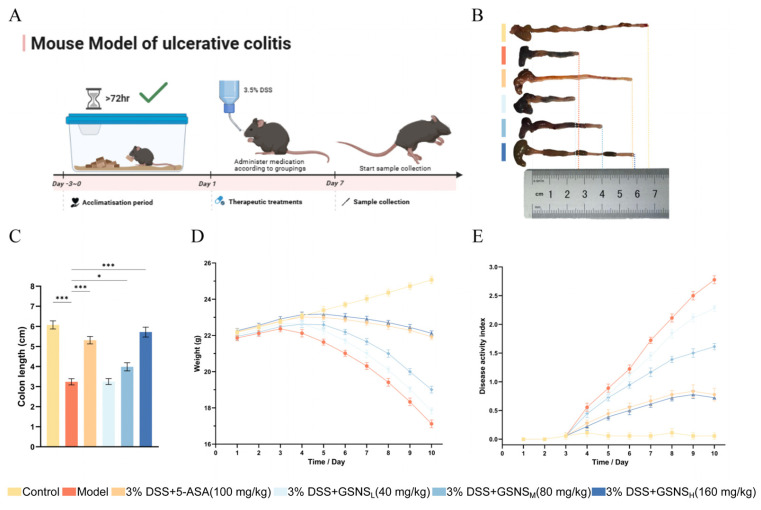
Therapeutic effects of GSNS on DSS-induced colitis mice. (**A**) Schematic diagram of the experimental design and treatment timeline. (**B**) Representative macroscopic photographs of colon tissues from each group. (**C**) Measurement results of colon length. (**D**) Body weight change curves during the treatment period. (**E**) DAI scores. Data are expressed as the mean ± SEM (*n* = 6). Statistical significance: * *p* < 0.05, *** *p* < 0.001 compared with the model group.

The experimental design and treatment timeline are summarized in Figure 2A, and no animals were excluded, with all data points included in the analysis. As shown in Figure 2B, gross examination of colon tissues revealed marked shortening and atrophy in the model group compared to the control. Quantitative measurement confirmed this observation, indicating that colon length in the model group was significantly reduced (*p* < 0.001, Figure 2C). Regarding dynamic disease progression, control mice exhibited steady weight gain throughout the experiment, while model group mice showed progressive weight loss from day 4 onward (Figure 2D). Concordantly, the DAI remained low in controls but rose sharply in the model group (Figure 2E). Intervention with the GSNS dose-dependently alleviated DSS-induced weight loss (*p* < 0.05, Figure 2D) and significantly reduced DAI scores in the high-dose group (*p* < 0.05, Figure 2E), with efficacy comparable to 5-ASA (*p* > 0.05). Furthermore, GSNS_M_ and GSNS_H_ treatments significantly reversed colon shortening (*p* < 0.001, Figure 2C), demonstrating a dose-dependent protective effect on colonic structure.

### 3.3. Modulation of Inflammatory and Oxidative Stress Biomarkers by GSNS in Serum and Colon

**Figure 3:**
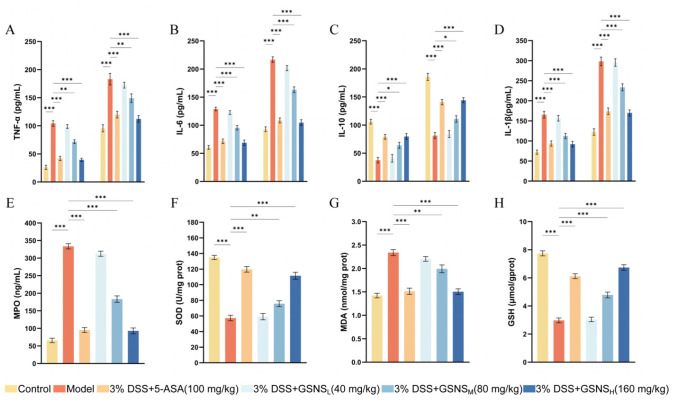
Modulation of inflammatory and oxidative stress profiles by GSNS in DSS-induced colitis mice. (**A**–**D**) Panels show GSNS effects on TNF-α, IL-6, IL-10, and IL-1β levels in both colon tissue and serum (*n* = 6). (**E**–**H**) Panels show GSNS effects on serum markers of oxidative stress: MPO, SOD, MDA, and GSH (*n* = 6). Statistical significance relative to the model group is indicated: * *p* < 0.05, ** *p* < 0.01, *** *p* < 0.001.

We confirmed the successful induction of the DSS-induced colitis mice based on a significant rise in pivotal pro-inflammatory cytokines [31]. As illustrated in Figure 3A–D, DSS induction resulted in a substantial increase (*p* < 0.001) in TNF-α, IL-6, and IL-1β levels in both serum and colon tissue. It also altered expression of IL-10, an anti-inflammatory cytokine. Administration of the GSNS and 5-ASA significantly reversed these aberrant cytokine profiles (*p* < 0.05, *p* < 0.01, or *p* < 0.001). The suppressive effect of the GSNS on pro-inflammatory cytokines was dose dependent, with the high-dose group exhibiting efficacy comparable to the 5-ASA group. Furthermore, data on colonic oxidative stress markers (Figure 3E–H) showed that the GSNS reduced MPO activity and MDA content in a dose-dependent manner while enhancing SOD activity and GSH levels. This antioxidant effect was most pronounced at GSNS_H_, equivalent to that achieved with 5-ASA treatment.

### 3.4. Histopathology of Colon Tissue and Immunohistochemical Analysis of Nrf2 Protein Expression

**Figure 4:**
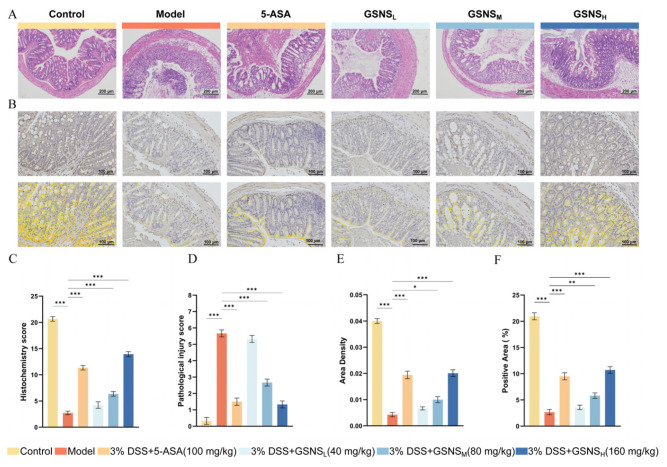
Histopathological evaluation and Nrf2 expression in colonic tissues of DSS-induced colitis mice treated with GSNS. (**A**) Representative H&E-stained sections of colon tissues (scale bar, 200 μm). (**B**) Representative immunohistochemical staining images showing Nrf2 expression in colon tissues (scale bar, 100 μm). (**C**) Histopathological injury scores. (**D**) Immunohistochemical scores for Nrf2 expression. (**E**) Quantitative analysis of the Nrf2-positive area ratio. (**F**) Quantitative analysis of the Nrf2-positive area density. * *p* < 0.05, ** *p* < 0.01, *** *p* < 0.001 compared with the model group. The results are expressed as mean ± SEM.

Colon tissue H&E staining results and pathological scores are shown in Figure 4A,C. The control group displayed preserved colonic architecture, characterized by an intact mucosal layer and regularly arranged glands. Inflammatory cells were only sporadically present in the lamina propria. In contrast, the model group displayed typical pathological features of severe UC: extensive mucosal epithelial defects, substantial depletion of goblet cells, destruction and loss of crypt architecture, severe submucosal oedema, and transmural infiltration by a large number of inflammatory cells (*p* < 0.001 vs. control group) [32]. GSNS treatment improved these pathological injuries in a dose-dependent manner. Compared with the model group, the GSNS_M_ and GSNS_H_ groups showed significantly reduced inflammatory infiltration and improved mucosal architecture (*p* < 0.001). Notably, the overall histopathological score in the GSNS_H_ group was not significantly different from that in the 5-ASA group (*p* > 0.05), indicating a similar degree of improvement.

The IHC results presented in Figure 4B,D–F demonstrate that, compared with the control group, the DSS model group exhibited a significantly reduced positive area ratio, areal density, and nuclear positive expression intensity (H-Score) of the Nrf2 protein in colon tissue (*p* < 0.001 for each). The decrease in the nuclear H-Score, in particular, indicated impaired nuclear translocation of Nrf2. Following GSNS intervention, the positive area ratio, areal density, and nuclear H-Score of Nrf2 increased in a dose-dependent manner in the GSNS_M_ and GSNS_H_ groups. The extent of recovery in the GSNS_H_ group was equivalent to that in the 5-ASA group, with no statistically significant difference [33,34]. These results confirm, at the tissue level, that the GSNS can effectively promote the expression and nuclear translocation of Nrf2 in colon tissue, thereby activating the Nrf2 signaling pathway.

### 3.5. Transmission Electron Microscopy Analysis of the Ultrastructure of the Colon Epithelium

**Figure 5:**
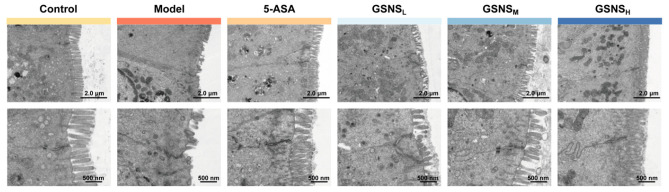
Effects of GSNS on colonic epithelial barrier integrity. Representative TEM images depicting the ultrastructure of colonic epithelial tight junctions.

Representative transmission electron micrographs of colonic tissue are shown in Figure 5. Examination by TEM revealed that control intestinal epithelial cells were characterized by dense, orderly microvilli, intact tight and gap junctions, and normal organelle structure. The DSS model group demonstrated severe ultrastructural damage: sparse or shed microvilli, widened intercellular spaces, blurred or absent tight junctions, and swollen, vacuolated mitochondria [35]. Following GSNS treatment, epithelial ultrastructure exhibited dose-dependent repair. In the GSNS_H_ group, intestinal epithelial microvillus density, cell junction integrity, and organelle status all approached normal levels, comparable to the 5-ASA group.

### 3.6. GSNS Enhances Antioxidant and Barrier Functions at the Genetic and Protein Levels

**Figure 6:**
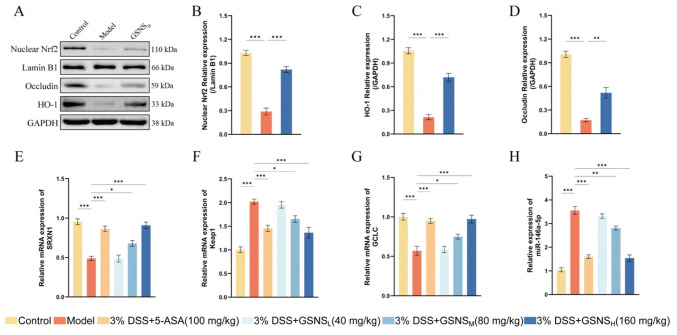
GSNS alleviated colitis and modulated the expression of associated proteins and genes in DSS-induced colitis mice. (**A**) Representative protein bands of Nrf2, HO-1, and Occludin (*n* = 3). (**B**–**D**) Densitometric analysis of nuclear Nrf2 (**B**), HO-1 (**C**), and Occludin (**D**) protein expression. (**E**) Relative mRNA expression of SRXN1. (**F**) Relative mRNA expression of Keap1. (**G**) Relative mRNA expression of GCLC. (**H**) Relative expression of miR-146a-5p. (**E**–**G**) Expression levels of mRNA genes SRXN1, Keap1, and GCLC in each group. (H) Expression level of the miRNA gene miR-146a-5p in each group. * *p* < 0.05, ** *p* < 0.01, *** *p* < 0.001 versus the model group. Data are expressed as mean ± SEM (*n* = 3).

Western blot analysis results for protein expression are presented in Figure 6A and corroborate the corresponding changes at the gene level. A marked reduction in the expression of Occludin, nuclear Nrf2, and its downstream antioxidant protein HO-1 was observed in the model group [36]. Following GSNS treatment, the protein expression levels of all three targets were significantly restored. Furthermore, densitometric analysis of protein bands (Figure 6B–D) revealed that, compared to the control group, the model group exhibited significantly reduced expression of nuclear Nrf2 (B), HO-1 (C), and Occludin (D) (all *p* < 0.001). GSNS_H_ treatment significantly reversed these reductions (*p* < 0.001 for nuclear Nrf2 and HO-1; *p* < 0.01 for Occludin). The RT-qPCR results (Figure 6E–H) indicated that DSS-induced colitis significantly upregulated Keap1 mRNA (Figure 6F) and miR-146a-5p (Figure 6H) expression in colon tissue (*p* < 0.001) while suppressing the expression of the anti-oxidant gene GCLC (Figure 6G) and the transcription factor SRXN1 (Figure 6E). GSNS intervention reversed these abnormal expressions in a dose-dependent manner, with the GSNS_H_ showing the most pronounced effects. In summary, this study demonstrates, at both transcriptional and translational levels, that the GSNS alleviates colitis by inhibiting Keap1, promoting Nrf2 nuclear translocation (as evidenced by increased nuclear Nrf2 protein levels), and subsequently upregulating downstream antioxidant protein HO-1 and barrier protein Occludin. Furthermore, its regulation of relevant miRNAs, such as miR-146a-5p, highlights its multi-level, synergistic mechanism of action.

### 3.7. Analysis of Gut Microbiota Changes Following GSNS Treatment

**Figure 7:**
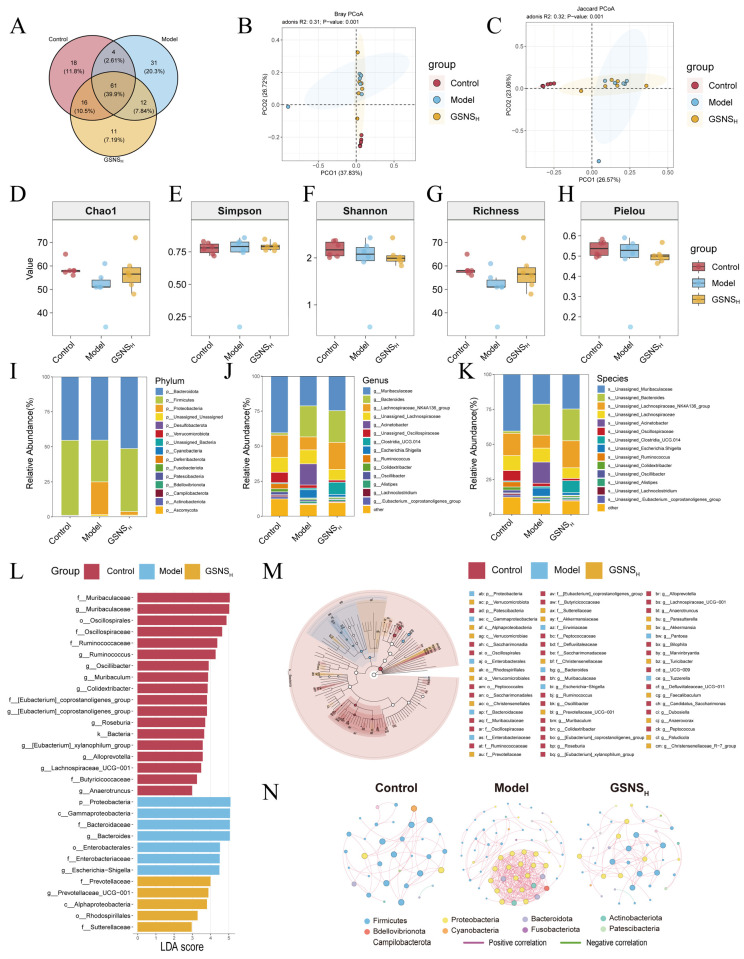
Analysis of intestinal contents via 16S rRNA gene sequencing reveals the modulatory effect of GSNS on DSS-induced microbiota dysbiosis. (**A**) Venn diagram of group-specific and shared ASVs; (**B**,**C**) PCoA based on Bray–Curtis and Jaccard distances. Alpha-diversity indices: (**D**) Chao1, (**E**) Simpson, (**F**) Shannon, (**G**) richness, and (**H**) Pielou’s evenness. Microbial community composition at the (**I**) phylum, (**J**) family, and (**K**) genus levels. (**L**) Histogram of LDA scores representing significantly discriminant taxa across groups. (**M**) Cladogram generated by LEfSe analysis illustrating the phylogenetic distribution of discriminant taxa. (**N**) Spearman correlation-based network analysis of microbial interactions. The results are expressed as mean ± SEM (*n* = 6).

The effect of GSNS on the gut microbiota in DSS-induced colitis mice was examined by 16S rRNA gene sequencing of cecal contents. A Venn diagram was generated to visualize OTU overlap, showing common and group-specific microbial features. The Venn diagram analysis revealed the compositional characteristics of OTU distribution across groups (Figure 7A). The proportions of unique OTUs in the control, model, and GSNS_H_ groups were 20.3%, 11.8%, and 7.19%, respectively, indicating that each group harbored a certain proportion of unique microbial taxa. Intergroup comparisons revealed the lowest OTU overlap between the control and model groups (2.61%), indicating that DSS modelling significantly altered the fundamental composition of the gut microbiota. The GSNS_H_ group showed 7.84% OTU overlap with the control group and 10.5% with the model group, suggesting that the microbiota structure after GSNS intervention retained features characteristic of both normal and diseased states. Furthermore, the OTUs shared among the three groups accounted for the highest proportion (39.9%), indicating a core microbiota that remains relatively stable under experimental conditions. These findings demonstrate that DSS intervention significantly alters microbial composition, while GSNS treatment partially restores structural features resembling those of normal microbiota. Assessment of microbial community structure differences between groups via beta-diversity analysis revealed significant segregation in Bray–Curtis and Jaccard distance principal coordinate analysis (Figure 7B,C) [37,38]. Samples from the GSNS intervention group exhibited distribution patterns closer to the normal control group. Further assessment of intra-group microbial diversity was performed via alpha-diversity analysis using multiple indices (Chao1, Shannon, Simpson, richness, and Pielou’s evenness; Figure 7D–H). The results consistently showed that DSS modeling significantly reduced both microbial richness and evenness in the gut. In contrast, GSNS treatment restored these diversity metrics in a dose-dependent manner, with the most pronounced recovery observed in the GSNS_H_ group. At the phylum level, DSS induction led to a decreased relative abundance of Firmicutes and an increase in Bacteroidetes, a trend that was reversed upon GSNS treatment. This community structure analysis across phylum, family, and genus levels is presented in bar charts (Figure 7I–K), where each bar represents one sample, colors denote different taxa, and the vertical axis shows relative abundance.

LEfSe was employed to identify species with significant intergroup differences (LDA score > 3.5) [39], and the results are visualized in Figure 7L,M. Across the four groups at the phylum and genus levels, a total of 30 discriminant taxonomic groups were identified: 18 in the control group, 7 in the model group, and 5 in the GSNS_H_ group. Key taxonomic units with significantly different abundances in the control group gut microbiota included Muribaculaceae, Oscillospirales, Oscillospiraceae, Ruminococcaceae, Ruminococcus, Oscillibacter, Muribaculum, Colidextribacter, the [*Eubacterium*] coprostanoligenes group (at both family and genus levels), and the genus *Roseburia*. Key taxonomic units significantly enriched in the gut microbiota of the model group included the phylum Proteobacteria, Gammaproteobacteria, Bacteroidaceae, *Bacteroides*, Enterobacterales, Enterobacteriaceae, and *Escherichia-Shigella*. Key taxonomic units significantly enriched in the GSNS_H_ group gut microbiota were Prevotellaceae, Prevotellaceae_UCG-001, and Alphaproteobacteria. The enrichment of these groups may reflect the specific regulatory effects of GSNS intervention on microbial community structure, and is associated with improvements in intestinal function.

Furthermore, Spearman correlation network analysis revealed alterations in microbial interactions. Results showed that, in the model group, gut microbiota exhibited a disordered interaction pattern centered on inflammation-associated groups such as Proteobacteria. Following GSNS intervention, the microbial network structure underwent significant remodeling; not only was positive synergy enhanced among beneficial groups like *Lactobacillus* and *Dubosella*, but their associations with opportunistic pathogens such as Enterobacteriaceae were also weakened. Concurrently, the interaction patterns of groups labeled “positively correlated” in Figure 7N, such as the phylum Cyanobacteria, became more stable. This further enhanced overall network complexity and structural stability. These findings indicate that the GSNS not only restores gut microbiota balance at the diversity and community structure levels but also enhances ecological network stability by reconfiguring beneficial microbial collaborations. This systemic approach supports the improvement of the pathological state of colitis.

## 4. Discussion

A DSS-induced mouse model of colitis was employed to evaluate the therapeutic potential and mechanistic basis of the GSNS. Integrated pharmacodynamic, molecular, and microbiome analyses demonstrated that the GSNS significantly alleviates disease severity. Its beneficial actions appear to be mediated through a multi-targeted mechanism involving suppression of systemic and local inflammation, activation of the Nrf2/HO-1 pathway to mitigate oxidative stress, enhancement of intestinal barrier integrity, and restoration of gut microbiota homeostasis. These findings collectively support that the GSNS exerts its protective effect via a coordinated modulation of the inflammation, oxidative stress, barrier, and microbiota axis.

In terms of anti-inflammatory and antioxidant effects, the pharmacodynamic findings of this study corroborate the mechanisms of action reported in the literature for the active components of individual herbs [16,17,18]. As shown in Figure 2, Figure 3 and Figure 4, GSNS intervention dose-dependently improved DSS-induced weight loss, colon length, and alleviated histopathological damage while significantly reducing levels of pro-inflammatory factors (TNF-α IL-6, IL-1β). These effects align with established reports that ginsenosides, salvianolic acids, and notoginsenosides suppress inflammation by modulating the NF-κB pathway [40,41,42]. Crucially, immunohistochemical and molecular biological analyses further revealed the GSNS’s direct antioxidant mechanism: it significantly promoted Nrf2 protein expression and nuclear translocation in colonic tissue (Figure 4) while upregulating the downstream antioxidant protein HO-1. This in situ evidence confirms that the GSNS effectively retains and integrates the core activity of its constituent herbs, bolstering cellular antioxidant defenses via activation of the Nrf2/HO-1 signaling axis. [43,44].

This research demonstrates that the GSNS, particularly at GSNS_H_, achieved comparable improvement levels to the 5-ASA group in key parameters, including DAI, colon length, and histopathological score. The comprehensive restorative effect observed in the whole animal model suggests potential synergistic interactions among the multiple components within this compound formulation [45,46,47]. The enhanced overall efficacy is likely due to the distinct active constituents of ginseng, salvia, and notoginseng acting on interrelated pathological pathways, such as inflammation, oxidative stress, and barrier repair, which ultimately converge to form a more robust, multi-target therapeutic network [48,49,50]. This amplifies the contributions of individual herbs and exemplifies the holistic regulatory characteristics of traditional Chinese medicine formulas.

At the molecular level, this study further revealed the regulatory effect of the GSNS on the Nrf2 signaling pathway. Enhanced nuclear translocation of Nrf2 was observed following GSNS treatment, along with upregulated expression of its downstream effector HO-1 and the tight junction protein Occludin in colon tissues, as determined by Western blot. These changes were consistent with transcriptional alterations: RT-qPCR results confirmed that GSNS downregulated Keap1 mRNA expression while upregulating antioxidant genes GCLC and Srxn1 [51,52,53]. Furthermore, the GSNS restored the abnormally elevated level of miR-146a-5p, a miRNA involved in inflammatory feedback regulation, in DSS-induced mice to near-normal levels [54]. These data suggest that the GSNS sustains activation of the Nrf2/HO-1 antioxidant axis by inhibiting Keap1 and may further facilitate inflammation resolution by regulating miR-146a-5p. Notably, the activation of antioxidant signaling and the enhancement of epithelial barrier function were synchronous and dose dependent, indicating that the GSNS coordinates key pathways such as Nrf2 to synergistically restore intracellular redox balance and physical barrier integrity.

To elucidate the regulatory effects of the GSNS on gut microbiota, 16S rRNA gene sequencing was performed in DSS-induced UC mice [55,56]. Venn analysis indicated a marked alteration in microbial composition following DSS induction, with only 2.61% of OTUs shared between the control and model groups. After GSNS intervention, the overlap with the control group increased to 7.84%, suggesting a partial restoration of microbial structure. Alpha-diversity indices, including Chao1 and Shannon, demonstrated that the GSNS dose-dependently recovered the DSS-induced reduction in microbial richness and evenness. Beta-diversity analysis further supported that GSNS treatment shifted the microbial community closer to that of the control group, as measured by Bray–Curtis and Jaccard distances. At the phylum level, the GSNS significantly mitigated the DSS-induced dysbiosis, particularly by restoring the Firmicutes/Bacteroidetes ratio. LEfSe analysis revealed that the GSNS downregulated pro-inflammatory taxa enriched in the model group, such as Proteobacteria, Enterobacteriaceae, and *Escherichia-Shigella*, while promoting beneficial genera including Prevotellaceae. Importantly, Spearman correlation network analysis showed that the GSNS remodeled microbial interactions: the Proteobacteria-centered network observed in the model group was replaced, positive correlations among beneficial bacteria such as *Lactobacillus* were strengthened, and overall network complexity and stability were enhanced. In summary, the GSNS not only rectifies compositional imbalances in the gut microbiota but also fosters a healthier ecological network, highlighting its role in restoring intestinal microenvironment homeostasis and showing its therapeutic potential in UC.

The findings of this study demonstrate that the GSNS alleviates colitis by synergistically activating the Nrf2/HO-1 antioxidant pathway, repairing epithelial tight junctions, and reshaping the gut microbiota. This provides an integrated therapeutic model with a clear multi-target mechanism for complex diseases such as UC, which are characterized by a vicious cycle of oxidative stress, barrier disruption, and dysbiosis [57,58,59]. The mode of action of the GSNS suggests that its components may act synergistically or through additive effects at the host–microbiota interface to restore intestinal homeostasis.

However, as a preclinical study, translation to human applications requires further validation [60]. Our experimental design lacked a vehicle control group (without DSS induction) and a GSNS-only group with healthy mice to rule out the non-specific effects or toxicity of the GSNS. The absence of a mesalamine-positive control group also prevents benchmarking against current clinical standards. Future studies incorporating these essential controls are necessary to validate the therapeutic potential of the GSNS. The optimal dosage, long-term safety, and pharmacokinetic profile of the GSNS in humans remain to be determined. Moreover, while synchronous improvements across pathways (such as the Nrf2/HO-1 axis) were observed, their temporal and causal relationships are not fully resolved. Future studies are needed to distinguish if microbiota alterations are primary or secondary to the therapeutic effects of the GSNS. The histological evaluation would have been more informative if immunohistochemical staining for specific immune cell markers such as MPO (neutrophils) or F4/80 (macrophages) could had been performed. Approaches such as using germ-free animals, performing fecal transplants, Nrf2 knockout models, or isolating specific constituents could help clarify these mechanistic nuances. In addition, the specific chemical constituents responsible for the antioxidant, anti-inflammatory, and microbiota-modulating effects of the GSNS need to be identified through compound isolation and reconstitution experiments [61]. Current analyses based on 16S rRNA sequencing primarily provide information on microbial community structure. A multi-omics approach encompassing metagenomics, metabolomics, and transcriptomics is warranted to delineate the specific interactions among microbial genes, metabolites, and host pathways, and to fully decipher the comprehensive regulatory network orchestrated by the GSNS.

In summary, this work systematically delineates the multi-target, multi-dimensional action framework of the GSNS in colitis, offering empirical support for the “multi-component–multi-target–multi-pathway” paradigm of herbal formulations in treating complex diseases. It lays a foundation for developing innovative strategies aimed at holistically restoring intestinal homeostasis. Future efforts should focus on identifying active components, elucidating mechanistic causality, and advancing clinical translation to provide UC patients with a potential therapy possessing systemic regulatory benefits.

## 5. Conclusions

In conclusion, this study demonstrates that the GSNS alleviates DSS-induced colitis through a multi-target mechanism. The GSNS exerts its effects by activating the Nrf2 signaling pathway to combat oxidative stress and inflammation, restoring intestinal barrier integrity via Occludin upregulation and preservation of tight junction ultrastructure (as confirmed by TEM). Additionally, treatment was associated with favorable modulation of gut microbiota homeostasis. Collectively, the therapeutic outcome involves the synergistic regulation of interconnected antioxidant, anti-inflammatory, barrier repair, and microbial modulating pathways. These findings provide modern pharmacological validation for the application of this traditional Chinese medicine formulation in DSS-induced colitis management, highlighting activation of the Nrf2 signaling pathway as a central mechanism underlying its multi-target efficacy.

## Figures and Tables

**Table 1:** Primer sequences used for gene expression analysis by RT-qPCR.

Gene	Primer Sequences (5′to 3′)		Fragment Size (bp)	Gene Accession Number
KEAP1	Forward	GAGATATGAGCCAGAGCGGGA	270	NM_001110305.1
	Reverse	AACTGGTCCTGCCCATCGTAG		
GCLC	Forward	CTGTAGATGATAGAACACGGGAGG	215	NM_010295.2
	Reverse	GAGATGAGCAACGTGCTGTGC		
Srxn1	Forward	GTACCAATCGCCGTGCTCAT	222	NM_029688.6
	Reverse	GAGCTTGGCAGGAATGGTCT		
Ywhaz	Forward	TTGTAGGAGCCCGTAGGTCATC	249	NM_001253805.1
	Reverse	CAGCAACCTCGGCCAAGTAA		
Hprt1	Forward	TCATGGACTGATTATGGACAGGACT	138	NM_013556.2
	Reverse	GCTTTAATGTAATCCAGCAGGTCAG		
miR-146a-5p	Forward	ACACTCCAGCTGGGTGAGAACTGAATTCCA	66	MIMAT0000158
	Reverse	TGGTGTCGTGGAGTCG		
U6	Forward	CTCGCTTCGGCAGCACA	94	NR_004394.2
	Reverse	AACGCTTCACGAATTTGCGT		

**Table 2:** Chemical compositions of GSNS.

NO	Name	Formula	Mass (Da)	Measured m/z	Error (ppm)	Rt (min)	Score	Polarity Mode
1	20(S)-Ginsenoside Ck	C_36_H_62_O_8_	644.42394	645.43283	2.5	73.558	80.1	Positive
2	Apigenin-7-O-β-D-glucoside	C_21_H_20_O_10_	432.10527	433.11289	0.8	38.479	85.2	Positive
3	Cimifugin	C_16_H_18_ O_6_	306.11	307.11748	0.65	58.765	83.4	Positive
4	Cryptotanshinone	C_19_H_20_O_3_	296.14109	297.14869	1.1	34.799	90.7	Positive
5	Ginsenoside Rk1	C_42_H_70_O_12_	766.48583	767.49351	0.52	70.74	88.8	Positive
6	Pseudoginsenoside F11	C_42_H_70_O_12_	800.49144	801.49965	1.17	71.467	81.2	Positive
7	Tanshinone IIA	C_19_ H_18_O_3_	294.12515	295.13271	0.96	58.234	90.9	Positive
8	20(R)-Ginsenoside Rg2	C_42_H_72_O_13_	784.49824	783.49190	1.2	61.71	84	Negative
9	20(R)-Notoginsenoside R2	C_41_H_70_O_13_	770.48215	769.47537	0.65	60.069	81.1	Negative
10	Chikusetsu saponin IVa	C_42_H_66_O_14_	794.44624	793.43976	1	72.926	87.4	Negative
11	Cynaroside	C_21_H_20_O_11_	448.09996	447.09327	1.3	39.605	80.6	Negative
12	Ginsenoside F1	C_36_H_62_O_9_	684.44514	683.43985	2.9	65.117	85.1	Negative
13	Ginsenoside F2	C_42_H_72_O_13_	830.50392	829.49897	2.8	75.861	87	Negative
14	Ginsenoside Rb1	C_54_H_92_O_23_	1108.60335	1107.59729	1.1	68.951	80.6	Negative
15	Ginsenoside Rb2	C_53_H_90_O_22_	1078.59237	1077.58779	2.5	69.6	80	Negative
16	Ginsenoside Rc	C_53_H_90_O_22_	1078.59237	1077.58768	2.4	71.066	81.9	Negative
17	Ginsenoside Rd	C_48_H_82_O_18_	945.54321	944.53745	1.6	73.504	82.4	Negative
18	Ginsenoside Re	C_48_H_82_O_18_	946.55049	945.54454	1.4	50.336	89.2	Negative
19	Ginsenoside Rf	C_42_H_72_O_14_	800.49283	799.48618	0.78	59.247	83.2	Negative
20	Ginsenoside Rg1	C_42_H_72_O_14_	846.49891	845.49290	1.5	63.744	81.3	Negative
21	Ginsenoside Rg2	C_42_H_72_O_13_	830.50435	829.49837	1.56	57.455	80.9	Negative
22	Ginsenoside Rg3	C_42_H_72_O_13_	784.4987	783.49282	1.78	75.298	86.7	Negative
23	Ginsenoside Ro	C_48_H_76_O_19_	956.49947	955.49317	1.02	69.874	80.4	Negative
24	Notoginsenoside Fe	C_47_H_80_O_17_	962.54677	961.54192	2.52	78.006	81	Negative
25	Notoginsenoside R1	C_47_H_80_O_18_	932.53523	931.52945	1.6	49.023	80.6	Negative

## Data Availability

The original contributions presented in this study are included in the article. Further inquiries can be directed to the corresponding authors.
